# Four New Jacaranone Analogs from the Fruits of a Beibu Gulf Mangrove *Avicennia marina*

**DOI:** 10.3390/md12052515

**Published:** 2014-04-30

**Authors:** Xiang-Xi Yi, Yong Chen, Wen-Pei Xie, Ming-Ben Xu, Yin-Ning Chen, Cheng-Hai Gao, Ri-Ming Huang

**Affiliations:** 1 School of Pharmaceutical Sciences, Guangxi University of Chinese Medicine, Nanning 530007, China; E-Mails: xiangxiyi81@aliyun.com (X.-X.Y.); 448614007@qq.com (Y.C.); xwpei-028@163.com (W.-P.X.); 2 Guangxi Key Laboratory of Marine Environmental Science, Guangxi Academy of Sciences, Nanning 530007, China; E-Mail: xumingben@gxas.cn; 3 Key Laboratory of Plant Resource Conservation and Sustainable Utilization, South China Botanical Garden, Chinese Academy of Sciences, Guangzhou 510650, China; E-Mail: chendianyu3356@163.com; 4 Department of Pharmacy and Pharmacology, University of Bath, Bath BA2 7AY, UK

**Keywords:** antioxidant, *Avicennia marina*, chlorocornoside, cornoside, jacaranone analogs, marinoid

## Abstract

Four new jacaranone analogs, marinoids F–I (**1**–**4**), were isolated from the fruits of a Beibu Gulf mangrove *Avicennia marina*. The structures were elucidated based on analysis of spectroscopic data. Marinoids F and G are shown to be diastereoisomers of chlorocornoside, a new halogen containing marine secondary metabolite. The antioxidant activity of the isolates was evaluated using a cellular antioxidant assay, and **4** showed good antioxidant activity (EC_50_ = 26 μM).

## 1. Introduction

**Figure 1:**
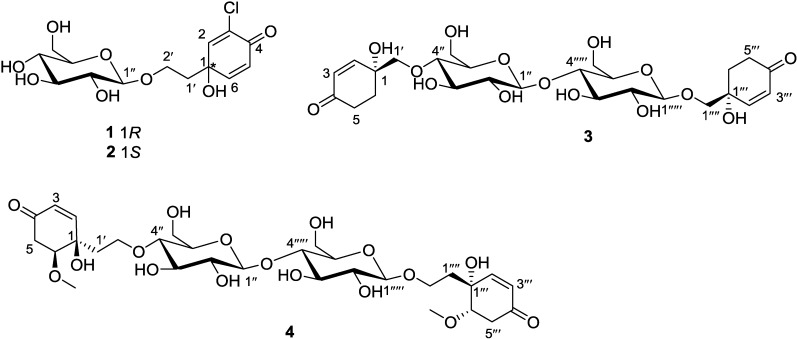
Structures of marinoids F–I (**1**–**4**).

*Avicennia marina* (Forsk.) Vierh. is commonly known as the grey or white mangrove plant resident in the tropical and subtropical regions, it is extremely widespread along the coasts of eastern Africa, islands of the Indian Ocean, tropical Asia, Australia, New Zealand, and islands of the Pacific Ocean to Fiji [1]. The crude extracts are reported to possess antimalarial and cytotoxic activities [1]. Different parts of the plant are used in Egypt as a folk medicine cure for skin diseases [2]. Previous chemical investigation of plants of the genus *Avicennina* have exhibited the presence of iridoid glucosides, marinoids A–E [1,2,3,4,5], naphthoquinone derivatives [6,7], flavonoids [4,8], and diterpenoids [9]. However, these previous studies did not report any chemical and biological data from the fruits of *A. marina*. Searching for bioactive secondary metabolites from this specimen afforded four new jacaranone analogs, marinoids F–I (**1**–**4** respectively) (Figure 1). In this paper, we describe the isolation, structural elucidation, and antioxidant activity of the four new secondary metabolites.

## 2. Results and Discussion

Marinoid F (**1**) was purified as a yellow oil with the molecular formula C_14_H_19_ClO_8_ as determined by HRESIMS (found [M + H]^+^ at *m/z* 351.0835, calcd [M + H]^+^, 351.0841) as well as ^1^H and ^13^C spectroscopic data (Table 1). ^1^H NMR spectra disclosed the presence of two methylene groups [(δ_H_ 3.94, ddd, *J* = 10.1, 7.0, 2.2 Hz, H-2′α) and 3.64, dd, *J* = 10.1, 2.2 Hz, H-2′β) and (δ_H_ 2.08, d, *J* = 7.0 Hz, H-1′)], an α,β-unsaturated carbonyl group [(UV λ_max_ 220 nm; δ_H_ 7.16 (d, *J* = 2.8 Hz, H-2); 6.99 (dd, *J* = 10.0, 2.8 Hz, H-6) and 6.22 (d, *J* = 10.0 Hz, H-5)]. The characteristic chemical shift of the carbonyl resonance (δ_C_ 179.3, C-4), in addition to the presence of four olefinic groups [δ_C_ 152.8 (C-6), 148.9 (C-2), 130.5 (C-3) and 125.8 (C-5)], and a quaternary sp^3^ carbon (δ_C_ 70.2, C-1) (Table 1), demonstrated that **1** has a *para*-quinol-type partial structure [10,11]. NMR spectra also indicated the presence of a β-glucosyl group, *i.e.*, one anomeric carbon resonance at δ_C_ 102.6 (C-1ʺ) and one anomeric proton at δ_H_ 4.18 (1H, d, *J* = 9.2 Hz, H-1ʺ). It was used as a starting point in the homonuclear correlated spectra to determine all glycosidic protons. The *J*_H-1ʺ-H-2ʺ_ value (9.2 Hz) of compound **1**, further confirmed that the sugar was a β-glucosyl group [12].

**Table 1:** ^1^H and ^13^C NMR data of marinoids F (**1**) and G (**2**) ^a^.

	1		2	
Position	δ_C_, Mult	δ_H_ (*J* in Hz)	δ_C_, Mult	δ_H_ (*J* in Hz)
1	70.2, C		70.2, C	
2	148.9, CH	7.16 (d, 2.8)	148.6, CH	7.20 (d, 2.8)
3	130.5, C		130.0, C	
4	179.3, C		179.1, C	
5	125.8, CH	6.22 (d, 10.0)	125.4, CH	6.19 (d, 10.0)
6	152.8, CH	6.99 (dd, 10.0, 2.8)	153.1, CH	7.03 (dd, 10.0, 2.8)
1′	39.6, CH_2_	2.08 (dd, 11.2, 7.0)	39.7, CH_2_	2.07 (dd, 11.2, 7.0)
2′α	63.9, CH_2_	3.94 (ddd, 10.1, 7.0, 2.2)	64.0, CH_2_	4.00 (dt, 10.4, 7.0, 2.4)
β		3.64 (dd, 10.1, 2.2)		3.65 (dd, 10.4, 2.4)
1ʺ	102.6, CH	4.18 (d, 9.2)	102.8, CH	4.21 (d, 7.8)
2ʺ	73.6, CH	3.13 (9.6, 9.2)	73.6, CH	3.14 (dd, 9.6, 7.8)
3ʺ	76.6, CH	3.20 (m)	76.6, CH	3.12 (m)
4ʺ	70.1, CH	3.24 (m)	69.9, CH	3.24 (m)
5ʺ	76.5, CH	3.29 (m)	76.6, CH	3.31 (m)
6ʺα	61.3, CH_2_	3.81 (dd, 11.9, 2.0)	61.4, CH_2_	3.84 (d, 11.8)
β		3.62 (m)		3.65 (m)

^a^ In CD_3_OD, 600 MHz for ^1^H and 150 MHz for ^13^C NMR.

**Figure 2:**
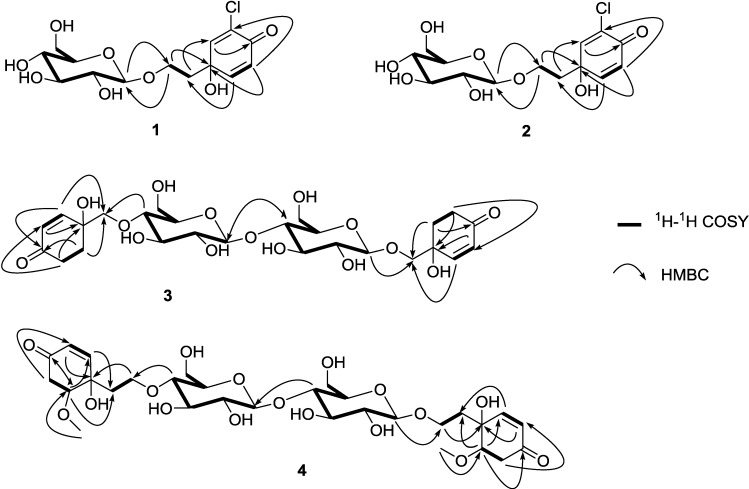
Selected ^1^H-^1^H COSY and HMBC correlations of marinoids F–I (**1**–**4**).

The gross structure was further established by the aid of COSY and HMBC experiments (Figure 2). A careful comparison of **1** and cornoside revealed that **1** differs from cornoside by the presence of one chlorine atom attached at C-3 [13]. Compound **1** showed 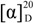 (MeOH) of −14.7°. The reported rotation value for cornoside is negative 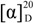 −10.5° [13], whose stereochemistry of the aglycone and the β-d-glucosyl residue have been established by enzymatic hydrolysis and other methods [13,14,15]. The reported rotation value after poly-acetylation of cornoside is also negative (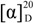 −10.2°) [16]. Moreover, the NMR data of the β-glucosyl residue in compound **1** are in accord with those observed in cornoside [13]. The substitution is simply that of one Cl atom for one H atom, and that many bonds away from the key chiral centre, therefore following those Literature data, we propose that the configuration of C-1 in compound **1** is the same as that found at C-1 in cornoside, namely *R*. Thus, the structure of **1** is predicted to be as shown in Figure 1.

Marinoid G (**2**) was obtained as yellow oil. Its molecular formula was determined as C_14_H_19_ClO_8_ by HRESIMS (found [M + H]^+^ at *m/z* 351.0837, calcd [M + H]^+^, 351.0841) as well as ^1^H and ^13^C data (Table 1). The NMR spectra of **2** are very similar to those of **1**. In addition, analysis of the COSY, HMBC and NOSEY correlations of **2** revealed identical spin systems and connections with those found in **1**. Compound **2** showed 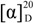 (MeOH) of +10.2°, and the reported value for cornoside (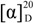 −10.5°) and poly-acetylated cornoside (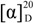 −10.2°) are both negative [13], and the observed value for **1** is −14.7°. Although we do not have an explanation for the differences in the absolute values of the optical rotations of cornoside, poly-acetylated of cornoside, and compound **1**, and the NMR data of the β-glucosyl residue in compound **2** are greatly similar to those of the β-glucosyl residue in cornoside and compound **1**, and also compound **2** showed a positive Cotton effect at 220 nm (Δε +5.97), whereas the observed value for **1** was −4.28, the opposite optical rotation and Cotton effect indicate that **1** and **2** are diastereoisomers. Indeed, we propose that they are enantiomers of the aglycone each with β-glucosyl residues.

Marinoid H (**3**) was obtained as a colourless oil and its molecular formula was established as C_26_H_38_O_15_ by HRESIMS (found [M + H]^+^ at *m/z* 591.2281, calcd [M + H]^+^, 591.2283) as well as ^1^H and ^13^C data (Table 2). In the NMR spectra of compound **3**, the proton signals at [δ_H_ 6.93 (1H, d, *J* = 15.1 Hz, H-2), 5.93 (1H, dd, *J* = 15.1, 2.6 Hz, H-3), 2.68 (1H, m, H-5α), 2.49 (1H, m, H-5β), 2.09 (1H, m, H-6α), and 2.03 (1H, m, H-6β)], and [δ_H_ 6.90 (1H, d, *J* = 15.1 Hz, H-2‴), 5.92 (1H, dd, *J* = 15.1, 2.6 Hz, H-3‴), 2.67 (2H, m, H-5‴), 2.08 (1H, m, H-6‴α), and 2.01 (1H, m, H-6‴β)] and the carbon signals at [δ_C_ 199.1 (C-4), 152.8 (C-2), 128.1 (C-3), 70.9 (C-1), 42.1 (C-5), and 37.3 (C-6)] and [δ_C_ 199.0 (C-4‴), 152.5 (C-2‴), 127.8 (C-3‴), 70.7 (C-1‴), 42.0 (C-5‴), and 37.2 (C-6‴)] indicated the existence of two cyclohexanone moieties [17], which were further confirmed by HMBC connections. NMR spectra also indicated the presence of two β-glucosyl groups by two anomeric carbon resonances at δ_C_ 102.9 (C-1ʺ) and 102.8 (C-1ʺ‴), and two anomeric protons at δ_H_ 4.27 (1H, d, *J* = 7.8 Hz, H-1ʺ) and 4.25 (1H, d, *J* = 7.8 Hz, H-1ʺ‴), which were further confirmed by the *J*_H-1ʺ__-__H-2ʺ_ and *J*_H-1__ʺ__‴__-__H-2__ʺ__‴_ values [18]. The tentative molecular weight of the compound deduced from NMR analysis suggested compound **3** to be an unsymmetrical dimer.

**Table 2:** ^1^H and ^13^C NMR data of marinoids H (**3**) and I (**4**) ^a^.

	3		4	
Position	δ_C_, Mult	δ_H_ (*J* in Hz)	δ_C_, Mult	δ_H_ (*J* in Hz)
1	70.9, C		72.4, C	
2	152.8, CH	6.93 (d, 15.1)	154.7, CH	6.95 (d, 10.0)
3	128.1, CH	5.93 (dd, 15.1, 2.6)	127.1, CH	5.87 (d, 10.0)
4	199.1, C		198.6, C	
5α	42.1, CH_2_	2.68 (m)	38.9, CH_2_	2.82 (dd, 11.1, 3.6)
β		2.49 (m)		2.49 (dd, 11.1, 7.0)
6α	37.3, CH_2_	2.09 (m)	82.8, CH	3.67 (dd, 7.0, 3.6)
β		2.03 (m)		
1ʹα	61.3, CH_2_	3.83 (d, 10.1)	34.4, CH_2_	2.18 (dd, 11.1, 5.3)
β		3.85 (d, 10.1)		2.00 (dd, 11.1, 6.1)
2ʹα			61.3, CH_2_	3.86 (d, 10.1)
β				3.79 (d, 10.1)
1ʺ	102.9, CH	4.27 (d, 7.8)	103.0, CH	4.28 (d, 7.5)
2ʺ	73.6, CH	3.13 (dd, 9.4, 7.8)	73.6, CH	3.14 (dd, 9.4, 7.8)
3ʺ	70.9, CH	4.08 (m)	70.2, CH	3.26 (m)
4ʺ	76.6, CH	3.32 (m)	76.7, CH	3.33 (m)
5ʺ	76.6, CH	3.25 (m)	76.7, CH	3.26 (m)
6ʺα	64.9, CH_2_	4.13 (dt, 9.5, 5.0)	65.1, CH_2_	4.16 (dt, 9.5, 5.0)
β		4.09 (dt, 9.5, 5.0)		4.14 (dt, 9.5, 5.0)
1‴	70.7, C		72.4, C	
2‴	152.5, CH	6.90 (d, 15.1)	154.0, CH	6.93 (d, 10.0)
3‴	127.8, CH	5.92 (dd, 15.1, 2.6)	127.0, CH	5.85 (d, 10.0)
4‴	199.0, C		198.6, C	
5‴α	42.0, CH_2_	2.67 (m)	38.9, CH_2_	2.82 (dd, 11.1, 3.6)
β				2.49 (dd, 11.1, 7.0)
6‴α	37.2, CH_2_	2.08 (m)2.01 (m)	82.7, CH	3.64 (dd, 7.0, 3.6)
β		2.01 (m)		
1ʺʺα	61.3, CH_2_	3.63 (d, 10.1)	34.3, CH_2_	2.17 (dd, 11.1, 5.3)
β		3.65(d, 10.1)		1.98 (dd, 11.1, 6.1)
2ʺʺα			61.3, CH_2_	3.84 (d, 10.1)
β				3.64(d, 10.1)
1ʺ‴	102.8, CH	4.25 (d, 7.8)	102.8, CH	4.27 (d, 7.5)
2ʺ‴	73.6, CH	3.13 (dd, 9.4, 7.8)	73.6, CH	3.14 (dd, 9.4, 7.8)
3ʺ‴	70.2, CH	3.25 (m)	70.2, CH	3.24 (m)
4ʺ‴	76.6, CH	3.31 (m)	76.6, CH	3.31 (m)
5ʺ‴	76.6, CH	3.31 (m)	76.6, CH	3.24 (m)
6ʺ‴α	64.9, CH_2_	3.78 (dt, 9.8, 5.5)	64.8, CH_2_	3.82 (dt, 9.8, 5.5)
β		3.78 (dt, 9.8, 5.5)		3.80 (dt, 9.8, 5.5)
OCH_3_			56.9, CH_3_	3.43 (s)
OCH_3_			56.9, CH_3_	3.43 (s)

^a^ In CD_3_OD, 600 MHz for ^1^H and 150 MHz for ^13^C NMR.

The gross structure was further established by the aid of COSY and HMBC experiments (Figure 2). Six spin systems could be revealed by analysis of COSY correlations corresponding to the H-2/H-3, H_2_-5/H_2_-6, H-1ʺ/H-2ʺ/H-3ʺ/H-4ʺ/H-5ʺ/H_2_-6ʺ, H-2‴/H-3‴, H_2_-5‴/H_2_-6‴, and H-1ʺ‴/H-2ʺ‴/H-3ʺ‴/H-4ʺ‴/H-5ʺ‴/H_2_-6ʺ‴. The connectivity of the two cyclohexanone moieties to C-1′ and C-1ʺʺ were established by the HMBC correlations of H-2 to C-1ʹ and H-6 to C-1ʹ, and of H-2‴ to C-1ʺʺ and H-6‴ to C-1ʺʺ, respectively. The presence of the two β-glucosyl groups in **3** could be proposed on the basis of HMBC correlations from H-4ʺ to C-1′ and H-1ʺ‴ to C-1ʺʺ, respectively. The connection of the two β-glucosyl groups was confirmed by the presence of HMBC correlation from H-4ʺ‴ to C-1ʺ. The configuration of C-1 and C-1‴ were assigned as *R* because a negative 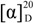 was observed, which was in accord with that observed in cornoside (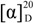 −10.5°), and analysis of the ^13^C NMR data of C-1 and C-1‴ in **3** indicated that they were greatly similar to that of C-1 (δ_C_ 68.9) in 4-[2-(β-d-glucopyranosyloxy)ethyl]-4-hydroxy-2-cyclohexen-1-one obtained from *Millingtonia hortensis*[19]. Consequently, the structure of **3** was determined as showed in Figure 1.

Marinoid I (**4**) was obtained as colourless oil. The presence of a molecular ion at *m/z* 701.2630 [M + Na]^+^ (calcd [M + Na]^+^, 701.2633) in the HRESIMS spectrum suggested a molecular formula of C_30_H_46_O_17_. Analysis of the ^1^H NMR data (Table 2) showed the presence of two cyclohexanone moieties in **4**[14] by the presence of the proton signals at δ_H_ 6.95 (1H, d, *J* = 10.0 Hz, H-2), 5.87 (1H, d, *J* = 10.0 Hz, H-3), 3.67 (1H, dd, *J* = 7.0, 3.6 Hz, H-6), 2.82 (1H, m, H-5α), and 2.49 (1H, m, H-5β)], and [δ_H_ 6.93 (1H, d, *J* = 10.0 Hz, H-2‴), 5.85 (1H, d, *J* = 10.0 Hz, H-3‴), 3.64 (1H, dd, *J* = 7.0, 3.6 Hz, H-6‴), 2.82 (1H, m, H-5‴α), and 2.49 (1H, m, H-5‴β), and the carbon signals at δ_C_ 198.6 (C-4), 154.7 (C-2), 127.1 (C-3), 72.4 (C-1), 82.8 (C-6), and 38.9 (C-5) and δ_C_ 198.6 (C-4‴), 154.0 (C-2‴), 127.0 (C-3‴), 72.4 (C-1‴), 82.7 (C-6‴), and 38.9 (C-5‴). The proton signals at δ_H_ 3.67 (1H, dd, *J* = 7.0, 3.6 Hz, H-6) and 3.64 (1H, dd, *J* = 7.0, 3.6 Hz, H-6‴), and corresponding carbon signals at δ_C_ 82.8 (C-6), 82.7 (C-6‴), 38.9 (C-5) and 38.9 (C-5‴) revealed that **4** was the CH_3_OH adduct of cornoside [13]. Moreover, the HMBC correlation from δ_H_ 3.43 (3H, s) to C-6 and δ_H_ 3.43 (3H, s) to C-6‴ we therefore assign the methoxyl to C-6 and C-6‴, respectively. NMR spectra also indicated the presence of two β-glucosyl groups, *i.e.*, two anomeric carbon resonances at δ_C_ 103.0 (C-1ʺ) and 102.8 (C-1ʺʺ), and two anomeric protons at δ_H_ 4.28 (1H, d, *J* = 7.5 Hz, H-1ʺ) and 4.27 (1H, d, *J* = 7.5 Hz, H-1ʺʺ). They were used as a starting point in the homonuclear correlated spectra to determine all the glycosidic protons. The *J*_H-1ʺ-H-2ʺ_ value (7.5 Hz) and of *J*_H-1ʺʺ-H-2ʺʺ_ value (7.5 Hz) compound **4**, further confirmed that the sugars were β-glucosyl groups [12]. The tentative molecular weight of the compound deduced from NMR analysis suggested compound **4** to be an unsymmetrical dimer of cornoside analog.

^1^H-^1^H COSY and HMBC correlations (Figure 2) were used to establish the molecular skeleton of **4**. Spin systems were revealed by analysis of COSY correlations corresponding to the H-2/H-3, H_2_-5/H-6, H_2_-1′/H_2_-2′, H-1ʺ/H-2ʺ/H-3ʺ/H-4ʺ/H-5ʺ/H_2_-6ʺ, H-2‴/H-3‴, H_2_-5‴/H-6‴, H_2_-1ʺʺ/H_2_-2ʺʺ, and H-1ʺ‴/H-2ʺ‴/H-3ʺ‴/H-4ʺ‴/H-5ʺ‴/H_2_-6ʺ‴. The connectivity of the two cyclohexanone moieties to C-1′ and C-1ʺʺ were established by the HMBC correlations of H-2 to C-1ʹ and H-6 to C-1ʹ, and of H-2‴ to C-1ʺʺ and H-6‴ to C-1ʺʺ, respectively. The presence of the two β-glucosyl groups in **4** could be proposed on the basis of HMBC correlations from H-4ʺ to C-2′ and H-1ʺ‴ to C-2ʺ, respectively. The connection of the two β-glucosyl groups was confirmed by the presence of HMBC correlation from H-4ʺ‴ to C-1ʺ.

The configuration of C-6, C-6‴, C-1 and C-1‴ were determined using the optical rotation and analysis of the coupling constants (*J*). The coupling patterns of the H-6 (dd, *J* = 7.0, 3.6 Hz) and H-5α (dd, *J* = 11, 3.6 Hz); H-6‴ (dd, *J* = 7.0, 3.6 Hz) and H-5‴α (dd, *J* = 11, 3.6 Hz) led to confirmation of the *cis*-orientation of H-6/H-5α and H-6‴/H-5‴α [20,21], which are in line with those of 4-[2-(β-d-glucopyranosyloxy)ethyl]-4-hydroxy-5-methoxy-2-cyclohexen-1-one that was obtained from *M. hortensis* [19], and 1,6-dihydroxy-4-oxo-2-cyclohexene-1-cetic acid ethyl ester isolated from *Senecio scandens* [17], and thus the configuration of C-6 and C-6‴ were determined as *S* and *S*. Analysis of the ^13^C NMR data of C-1 and C-1‴ in **4** indicated that they were greatly similar to that of C-1 (δ_C_ 71.8) in 4-[2-(β-d-glucopyranosyloxy)ethyl]-4-hydroxy-5-methoxy-2-cyclohexen-1-one [19], and our compound **4** showed 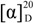 in MeOH of −26.4°, which is in accord with that observed in 1,6-dihydroxy-4-oxo-2-cyclohexene-1-acetic acid ethyl ester (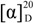 −12.5°) [17]. From the aforementioned analyses, the configurations of **4** were assumed to be 1*R*, 6*S*, 1‴*R* and 6‴*S*. On the basis of this cumulative analysis, the structure of **4** was thus determined as shown in Figure 1.

The cellular antioxidant assay (CAA) is a new approach to quantify antioxidants under physiological conditions when compared to chemical antioxidant activity assays [22,23,24]. The CAA assay has been widely used for fruits and vegetables recently, but not yet in marine natural products research. The EC_50_ values of compounds **1**–**3** were weak, 598, 4971, and 1103 μM, respectively. However, the EC_50_ value of compound **4** was 26 μM, of the same order of the positive control quercetin (EC_50_ = 11 μM).

## 3. Experimental Section

### 3.1. General Experimental Procedures

UV spectra were recorded in MeOH on a Perkin-Elmer Lambda 35 UV-Vis spectrophotometer (Wellesley, MA, USA). The IR spectra were measured in KBr on a WQF-410 FT-IR spectrophotometer (Beifen-Ruili, Beijing, China). NMR spectra were recorded on a Bruker AV 600 NMR spectrometer (Bruker, Bremen, Germany) with TMS as an internal standard. HR-ESI-MS data were obtained from Bruker Maxis mass spectrometer (Bruker, Bremen, Germany). Waters-2695 HPLC system (Waters, Milford, MA, USA), using a Sunfire™ C_18_ column (150 × 10 mm i.d., 10 μm, Waters, Milford, MA, USA) coupled to a Waters 2998 photodiode array detector (Waters, Milford, MA, USA). Optical rotation data were measured by Perkin-Elmer Model 341 polarimeter (Wellesley, MA, USA). CD spectra were recorded on a spectropolarimeter (MODEL J-810-150S, Tokyo, Japan). The silica gel GF_254_ used for TLC were supplied by the Qingdao Marine Chemical Factory, Qingdao, China. Spots were detected on TLC under UV light or by heating after spraying with 5% H_2_SO_4_ in EtOH. All solvent ratios are measured v/v.

### 3.2. Plant Material

The fruits of *A. marina* were collected from Beihai city, Guangxi province, China, in September, 2011. The specimen was identified by Professor Hangqing Fan who is from Guangxi Mangrove Research Center, Guangxi Academy of Sciences. A voucher specimen (2011-GXAS-008) was deposited in Guangxi Key Laboratory of Marine Environmental Science, Guangxi Academy of Sciences, China.

### 3.3. Extraction and Isolation

The fruits of *A. marina* (35.4 kg, wet weight) were exhaustively extracted with EtOH-CH_2_Cl_2_ (2:1, v/v). The solvent was evaporated *in vacuo* to afford a syrupy residue that was suspended in distilled water and fractionated successively with petroleum ether, ethyl acetate, and *n*-butanol. The *n*-butanol soluble portion (269 g) was subjected to column chromatography (CC) on silica gel, using CHCl_3_–MeOH (from 10:0 to 0:10) as eluent, giving eleven fractions (A–K). Fraction D was subjected to column chromatography to afford four subfractions (D1–D4). Fraction D3 was separated by HPLC, using MeOH–H_2_O (MeOH:H_2_O = 15:85, 25:75, 60:40) to yield **4** (3.9 mg, R_t_ = 10.2 min), **1** (3.5 mg, Rt = 12.5 min) and **2** (2.3 mg, R_t_ = 13.9 min), respectively. Fraction D4 was separated by HPLC, using MeOH–H_2_O (MeOH:H_2_O = 5:95) to yield **3** (6.0 mg, R_t_ = 9.5 min).

Marinoid F (**1**): Yellow oil; UV (MeOH) λ_max_ (log ε_max_) 220 (2.45) and 242 (3.31) nm. 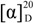 −14.7° (c 0.18, MeOH); CD (MeOH) Δε_220__nm_ −4.28, IR (KBr) ν_max_ 3425, 1720 and 1682 cm^−^^1^. ^1^H (CD_3_OD, 600 MHz) and ^13^C (CD_3_OD, 150 MHz) NMR data, see Table 1; HRESIMS: *m*/*z* 351.0835 (calcd. for C_14_H_19_ClO_8_ + H, 351.0841).

Marinoid G (**2**): Yellow oil; UV (MeOH) λ_max_ (log ε_max_) 220 (2.39) and 240 (2.75) nm. 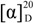 +10.2° (c 0.21, MeOH); CD (MeOH) Δε_220__nm_ +5.97; IR (KBr) ν_max_ 3424, 1711 and 1684 cm^−^^1^. ^1^H (CD_3_OD, 600 MHz) and ^13^C (CD_3_OD, 150 MHz) NMR data, see Table 1; HRESIMS: *m*/*z* 351.0837 (calcd. for C_14_H_19_ClO_8_ + H, 351.0841).

Marinoid H (**3**): Colourless oil; UV (MeOH) λ_max_ (log ε_max_) 218 (2.35) and 237 (2.94) nm. 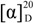 −15.8° (c 0.25, MeOH); CD (MeOH) Δε_220__nm_ −6.32; IR (KBr) ν_max_ 3452, 1701 and 1675 cm^−^^1^. ^1^H (CD_3_OD, 600 MHz) and ^13^C (CD_3_OD, 150 MHz) NMR data, see Table 1; HRESIMS: *m*/*z* 591.2281 (calcd. for C_26_H_38_lO_15_ + H, 591.2283).

Marinoid I (**4**): Colourless oil; UV (MeOH) λ_max_ (log ε_max_) 219 (2.15) and 238 (3.05) nm. 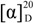 −26.4° (c 0.31, MeOH); CD (MeOH) Δε_220__nm_ −5.42; IR (KBr) ν_max_ 3447, 1705 and 1679 cm^−^^1^. ^1^H (CD_3_OD, 600 MHz) and ^13^C (CD_3_OD, 150 MHz) NMR data, see Table 1; HRESIMS: *m*/*z* 701.2630 (calcd. for C_30_H_46_O_17_ + Na, 701.2633).

### 3.4. Cellular Antioxidant Assay

Following the reported method [21,22,23], the cellular antioxidant activity was determined.

## 4. Conclusions

In conclusion, four new jacaranone analogs, marinoids F–I (**1**–**4** respectively), were isolated from a Beibu Gulf mangrove *A. marina* and identified. Marinoids F and G are shown to be diastereoisomers of chlorocornoside, a new halogen containing marine secondary metabolite. The CAA assay is considered to be a more physiologically relevant assay in the measurement of antioxidant activity of food when compared to the common chemistry antioxidant activity assays [25,26]. Until today, there have been no reports of the use of this CAA assay in the marine research area. Using the assay, the antioxidant activity of the isolates was therefore determined. This is the first report of chlorocornoside and of the dimeric disaccharide **4** which showed good antioxidant activity (EC_50_ = 26 μM), comparable with the positive control quercetin.

## References

[1] Sun Y., Ouyang J., Deng Z.W., Li Q.S., Lin W.H. (2008). Structure elucidation of five new iridoid glucosides from the leaves of *Avicennia marina*. Magn. Reson. Chem..

[2] Fauvel M.T., Taoubi K., Gleye J., Fourasté I. (1993). Phenylpropanoid glycosides from *Avicennia marina*. Planta Med..

[3] Konig G., Rimpler H. (1985). Iridoid glucosides in *Avicennia marina*. Phytochemistry.

[4] Feng Y., Li X.M., Duan X.J., Wang B.G. (2006). Iridoid glucosides and flavones from the aerial parts of *Avicennia marina*. Chem. Biodivers..

[5] Fauvel M.T., Bousquetmelou A., Moulis C., Gleye J., Jensen S.R. (1995). Iridoid glucosides from *Avicennia germinans*. Phytochemistry.

[6] Ito C., Katsuno S., Kondo Y., Tan H.T.W., Furukawa H. (2000). Chemical constituents of *Avicennia alba*. Isolation and structural elucidation of new naphthoquinones and their analogues. Chem. Pharm. Bull..

[7] Han L., Huang X.S., Dahse H.M., Moellmann U., Fu H.Z., Grabley S., Sattler I., Lin W.H. (2007). Unusual naphthoquinone derivatives from the twigs of *Avicennia marina*. J. Nat. Prod..

[8] Sharaf M., El-Ansari M.A., Saleh N.A.M. (2000). New flavonoids from *Avicennia marina*. Fitoterapia.

[9] Han L., Huang X.S., Dahse H.M., Moellmann U., Grabley S., Lin W.H., Sattler I. (2008). New abietane diterpenoids from the mangrove *Avicennia marina*. Planta Med..

[10] Parker K.A., Kang S.K. (1980). Regiospecific nucleophilic aromatic-substitution—Conjugate addition of active methylene-compounds to quinone monoacetals and aromatization of the adducts. J. Org. Chem..

[11] Hollenst R., Philipsb W.V. (1972). Carbon magnetic-resonance spectra of α, β, γ, σ- and α, β, α′, β′-unsaturated-ketones. Helv. Chim. Acta.

[12] Breitmaier E., Voelter W. (1987). Carbon-13 NMR Spectroscopy.

[13] Endo K., Hikino H. (1984). Structures of rengyol, rengyoxide, and rengyolone, new cyclohexylethane derivatives from *Forsythia suspensa* fruits. Can. J. Chem..

[14] Bianco A., Scalzo R.L., Scarpati M.L. (1993). Isolation of cornoside from *Olea europaea* and its transformation into halleridone. Phytochemistry.

[15] Jensen S.R., Kjaer A., Nielsen B.J. (1973). A quinol glucoside isolated from *Cornus* species. Acta Chem. Scand..

[16] Sasaki H., Taguchi H., Endo T., Yosioka I. (1978). The glycosides of *Martynia louisiana* Mill. A new phenylpropanoid glycoside, martynoside. Chem. Pharm. Bull..

[17] Tian X.Y., Wang Y.H., Yang Q.Y., Yu S.S., Fang W.S. (2009). Jacaranone analogs from *Senecio scandens*. J. Asian Nat. Prod. Res..

[18] Seo S., Tomita Y., Tori K., Yoshimura Y. (1978). Determination of the absolute configuration of a secondary hydroxy group in a chiral secondary alcohol using glycosidation shifts in carbon-13 nuclear magnetic resonance sepctroscopy. J. Am. Chem. Soc..

[19] Hase T., Kawamoto Y., Ohtani K., Kasai R., Yamasaki K., Picheansoonthon C. (1995). Cyclohexylethanoids and related glucosides from *Millingtonia hortensis*. Phytochemistry.

[20] Huang R.M., Ma W., Dong J.D., Zhou X.F., Xu T.H., Lee K.J., Yang X.W., Xu S.H., Liu Y.H. (2010). A new 1,4-diazepine from South China Sea marine sponge *Callyspongia* species. Molecules.

[21] Wang Y.L., Li Z.L., Zhang H.L., Sha Y., Pei Y.H., Hua H.M. (2008). New germacrane sequiterpenes from *Salvia chinensis*. Chem. Pharm. Bull..

[22] Faller A.L.K., Fialho E., Liu R.H. (2012). Cellular antioxidant activity of Feijoada whole meal coupled with an *in vitro* digestion. J. Agric. Food Chem..

[23] Song W., Derito C.M., Liu M.K., He X.J., Dong M., Liu R.H. (2010). Cellular antioxidant activity of common vegetables. J. Agric. Food Chem..

[24] Wolfe K.L., Kang X.M., He X.J., Dong M., Zhang Q.Y., Liu R.H. (2008). Cellular antioxidant activity of common fruits. J. Agric. Food Chem..

[25] Wolfe K.L., Liu R.H. (2007). Cellular antioxidant activity (CAA) assay for assessing antioxidants, foods, and dietary supplements. J. Agric. Food Chem..

[26] Felice D.L., Sun J., Liu R.H. (2009). A modified methylene blue assay for accurate cell counting. J. Funct. Foods.

